# Water conflicts under climate change: Research gaps and priorities

**DOI:** 10.1007/s13280-024-02111-7

**Published:** 2025-01-31

**Authors:** Elisie Kåresdotter, Georgia Destouni, Richard B. Lammers, Marko Keskinen, Haozhi Pan, Zahra Kalantari

**Affiliations:** 1 Department of Physical Geography and Bolin Centre for Climate Research, Stockholm University, Inst för Naturgeografi, 106 91, Stockholm, Sweden; 2 Earth Systems Research Center, Institute for the Study of Earth, Oceans, and Space, University of New Hampshire, Morse Hall, 8 College Road, Durham, NH 03824 USA; 3 Water & Development Research Group, Department of Built Environment, Aalto University, Aalto University Foundation Sr, PO BOX 11000, 00076 Aalto, Finland; 4 School of International and Public Affairs & China Institute for Urban Governance, Shanghai Jiao Tong University, Xin Jian Building, No. 1954 Huashan Rd, Shanghai, China; 5 Department of Sustainable Development, Environmental Science and Engineering (SEED), KTH Royal Institute of Technology, Kungliga Tekniska högskolan, SEED, 100 44 Stockholm, Sweden

**Keywords:** Conflict drivers, Climate–water–conflict nexus, Hydropolitics, Resource conflict, Scoping review

## Abstract

Climate change is known to worsen conflicts, but its combination with other factors affecting water-related conflicts remains less explored. Using a scoping review, this study examined research in the climate–water–conflict nexus. Using semi-automatic text mining approaches, key research gaps and differences in conflict factors and themes across different regions and conflict types were analyzed. Studies focused on Asia and Africa, with few exploring other regions. Governance and livelihoods emerged as significant factors in water-related conflict responses worldwide, with differences across regions. For instance, farmer–herder conflicts were common in Africa, while agriculture was more related to governance and water management in Asia. Research priorities forward should diversify the range of water-related conflict subjects and regions and give special focus to regions vulnerable to hydroclimatic change. More focus on cooperation and non-violent conflicts is also vital for understanding and being able to project and mitigate future water-related conflict responses to climate change.

## Introduction

Climate change is exacerbating water scarcity and water stress issues, affecting dry areas of the world more strongly and increasing the risks of water conflicts (Ide et al. 2021; Xia et al. 2021; Unfried et al. 2022). Conflicts are, in turn, increasing these impacts as adaptive capacities are decreased, with increased vulnerability from double exposure to both conflict and climate change impacts (Okpara et al. 2017; Muzamil et al. 2021). Water conflict in this study is defined as a disagreement leading to non-violent or violent action between at least two parties centered around freshwater, such as conflicts within or between countries on managing and distributing freshwater resources. Conflicts of multiple scales and severity are included, ranging from non-violent disputes between a few individuals to violent riots and, most severely, even though uncommon, wars between countries. Water-related risks of climate change have numerous security implications, including the potential for water conflicts, but also other issues, such as food security, increased incidence of various diseases (e.g., relating to access to clean water, increasing pathogen load, and spread with increased temperature), and increased probability of natural disasters such as drought, flash floods, and wildfires (Scheffran and Battaglini 2011). The discussion in this emerging climate–water–conflict nexus has reached a consensus that climate change generally acts as a threat multiplier rather than a driver of conflict (Scheffran and Battaglini 2011; Mach et al. 2019; Ide et al. 2021). Additionally, the discussion revolves around whether other drivers, such as political and socioeconomic factors and previous conflicts, may significantly impact the conflict outcomes (Theisen 2012; Dinar et al. 2015; Ide et al. 2021), with the involved conflict factors varying between regions due to their various hydroclimatic, socioeconomic, political, and cultural contexts (Dinar et al. 2015; Abel et al. 2019; Ide et al. 2020; von Uexkull and Buhaug 2021). Previous studies have largely focused on specific regions, with little comparison between regions, despite indications that drivers of water-related conflicts are region and context-dependent. Understanding the differences in conflict factors behind various conflict types and regions is important to be able to mitigate and strive toward limiting future conflicts. With water conflicts increasing in many parts of the world toward the present time (Kåresdotter et al. 2023), an updated perspective providing a regional understanding of water conflicts and their factors of importance can provide improved understanding and guidance for future research. An example of an indirect climate change pathway to water conflict is the Syrian conflict that started in 2011 and has been suggested to be partly triggered by climate change, with related problems of water scarcity, water management, and drought leading to large-scale rural-to-urban migration of farmers in the hope of finding better economic opportunities, and in turn, leading to social problems of large unemployment and overcrowding, and eventually conflict (Gleick 2014; Ide 2018; Abel et al. 2019). Drought has also been used to describe farmer–herder conflicts in Africa, where herders are increasingly coming into conflict with farmers due to increased difficulty finding adequate pasture, which can also be attributed to climate change (decreased precipitation) along with agricultural expansion (Fjelde and von Uexkull 2012; Brottem 2016). Loss of livelihood without adequate options, such as compensations for lost crops, and the inability of governments to manage the related challenges are important factors in shaping the pathways to these and similar conflicts (Fjelde and von Uexkull 2012; Ide et al. 2021). Historically, cooperation around water has been a more common outcome than conflict when facing water issues (Bernauer and Böhmelt 2020; Kåresdotter et al. 2023).

With increased pressure on water availability, affected both by changing hydroclimate and extended and/or intensified human activities (Kåresdotter et al. 2022), and an observed trend toward increased water conflicts (Kåresdotter et al. 2023), it is essential to understand the various underlying drivers and their interconnections that may combine to increase or decrease the water conflict risk, in order to address the challenges societies are facing in a way that can promote cooperation and peace. In particular, a need for joint understanding and simultaneous response to both climate change and conflict situations in various regions has been highlighted (Morales-Muñoz et al. 2022). This paper aims to investigate the global and regional factors that have shaped water conflicts affected by climate change in the past. Building upon the increased interest in the field and bridging the understanding between different regions, this review will improve the current understanding of the factors researched, how they vary between different regions, and conflict types (internal or transboundary conflicts). Further, this study will map the spatial distribution of previous studies, revealing variations in research interests across the globe. Lastly, the study will give suggestions for future research attention, including potential avenues for regions less researched based on study findings. With climate change generally considered a threat multiplier and not the driver of conflict, the hypothesis is that water-related conflicts will also depend on various other factors beyond climate change that combine with the latter to shape the conflicts.

The study aims and hypothesis testing is addressed through a scoping literature review for identifying and quantifying the degree to which the scientific literature covers various concepts and gaps relating to water conflict research. A scoping review is typically performed to map the extent of the existing literature and related key concepts and/or identify research gaps relating to a topic or research question (Arksey and O’Malley 2005; Peters et al. 2015). With a growing number of articles spanning multiple disciplines, manual screening of studies is becoming more and more time-consuming. As such, computational semi-automatic text analysis methods (text mining) were used in this study to help bridge the gap between disciplines and allow for multidisciplinary evaluation of the targeted water conflict topic that spans multiple disciplines and methods (Banks et al. 2018). Despite the potential and reliability of more automated text analysis (O’Mara-Eves et al. 2015), this has rarely been used to review previous research. The main research questions addressed were: Are there some key differences and similarities in studies of climate-affected water-related conflict in various parts of the world? What factors (e.g., parts of the world examined, drivers selected, geographical scales investigated) influence the possible discrepancies in conclusions between these conflict studies? These questions were investigated by sub-dividing relevant studies based on regions and spatial scales considered (from global to local). This division enabled the identification of the most studied regional contexts, how they vary across the globe, and important global patterns of studies.

## Materials and methods

### Screening

A semi-automated text mining approach was utilized, a method that has been shown to be largely reliable for screening with limited loss of relevant articles (O’Mara-Eves et al. 2015). First, a number of search queries were performed for relevant terms in titles, abstracts, and keywords, and results were extracted into CSV files using Scopus. The list was then exported to MATLAB, where different queries were performed, again on titles, abstracts, and keywords, to determine which studies were relevant for inclusion. The code used to speed up the screening process can be found in Zenodo. In short, the code was used to locate various conflict and water-related words and exclude articles related to wildlife, healthcare, and fisheries, as these topics are likely irrelevant to freshwater conflicts between humans.

**Fig. 1:**
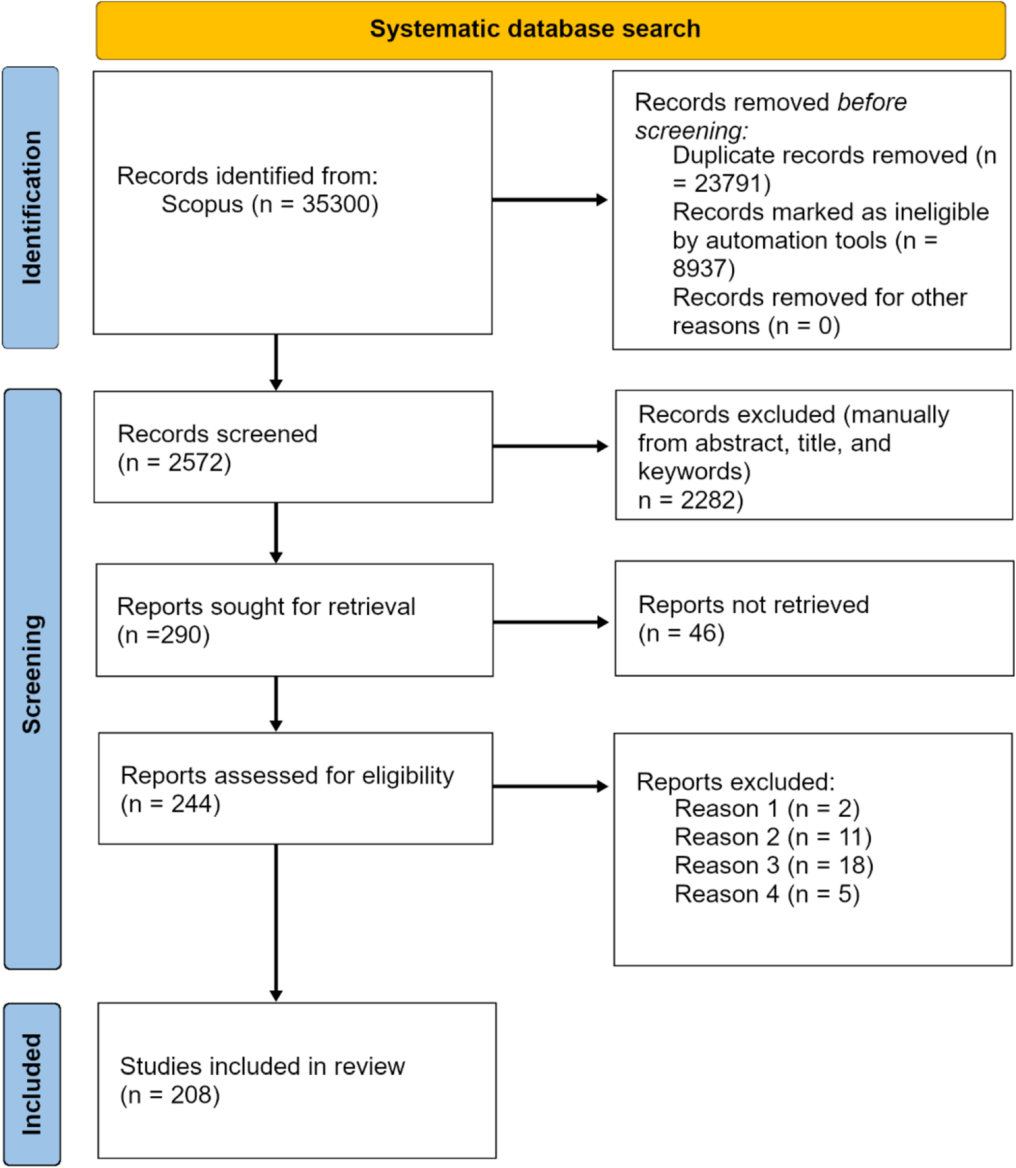
PRISMA flow diagram adapted from Page et al. (2021). Reason 1–3 follows inclusion criteria (i–iii), in short: (i) water-related conflict, (ii) climate change context, and (iii) historic or ongoing conflict. Reason 4) The study was in a useable format for text analysis extraction

The literature search was performed on 9 January 2024 using SCOPUS and was limited to studies written in English up until the year 2023, excluding books, editorials, and erratum. Search terms used were (1) (“climate change” OR “global warming” OR “environmental change”) AND (conflict OR dispute) AND (water OR hydrology), (2) (conflict OR dispute) AND hydroclimat*, (3) (“water conflict” OR “water dispute”) AND (precipitation OR temperature OR drought) (4) (conflict OR dispute) AND “climate change,” (5) hydropolitic*, and (6) “water conflict” OR “water dispute,” leading to a total of 11 509 matches (not counting duplicates). The final selection for the scoping review was made to include papers that discuss previous or ongoing water-related conflicts where climate change is a factor, meaning that articles discussing future conflict risks or conflicts that are not at least partly water-related were excluded. The three selection criteria that all needed to be fulfilled for each selected study were: (i) The study is considering water-related conflicts (conflicts directly or indirectly related to freshwater aspects, such as water scarcity or lack of access to water); (ii) the study considers the water-related conflict(s) in a climate change context (directly or indirectly related to climate change, including direct impact of climate change adaptation measures); and (iii) the study addresses factors relating to a previous or ongoing act of conflict (violent or non-violent) and not just potential conflicts or conflict mitigation measures, for example for future water resource decline. Using these criteria, the number of considered studies was semi-automatically filtered down to 2572 by screening the titles, abstracts, and keywords, and by further screening of the full texts, the final number of considered studies came down to 208 (Fig. 1).

### Semi-automatic textual analysis and spatial identification of studies

Using MATLAB, text from all studies was extracted and processed to form the baseline for analyzing the key characteristics (i.e., closely related terms). All studies were imported and preprocessed to extract text (without references, headlines, and author declaration of no conflict of interest) that could later be used for this analysis. Footnotes or references not easily removed using search criteria (such as “Reference” and “Literature cited”) were retained (11 studies), as they were estimated to have little impact on the results. The computational text analysis was structured into main categories to represent the broad range of concepts considered in the climate–water–conflict nexus and evaluate the factors addressed and their differences across continents (Table 1). A complete list of all words included in each category and references to all articles are available in Zenodo. To analyze statements about conflict and what could be driving them, words and word pairs within 100 characters (both directions) of the terms “conflict,” “dispute,” “competition,” “war,” “driver,” “drive,” “ignite,” and “trigger” were extracted from each article as a basis for the text analysis. Only using parts of the study texts means that the analyzed text is largely relevant to the investigated topic. Stop words like “to” or “in” were removed, and extracted words were lemmatized so that, e.g., the search word “lead conflict” would have the same meaning as, for example, lead to conflict or leading to conflict. To avoid double counting, overlap between extracted texts was merged. Plots and chord diagrams were then created using the extracted texts, either for all studies together or based on their allocated continent using the groups and categories (Table 1), by (1) calculating the number of times the terms of a category were mentioned (plots) or the number of times two categories were mentioned in the same context in the same study (chords); and (2) normalizing the absolute term number by dividing it with the associated number of studies (e.g., number of studies for Africa) for relative importance comparison between regions with different number of studies. A resulting category score of 1 means that all studies mentioned that category (plots) or two co-occurring categories (chords) on average one time in each study for the cleaned text (study text without title, authors, references, and author declaration of no conflict of interest).

**Table 1:** Categories and groups of categories used to structure the text analysis

Group	Category	Examples of terms included
Political	Governance	policy, politic*, govern*, federal
	Political instability	undemocra*, political unrest, corrupt*
	Economics	economy, finance, GDP, growth shock*
	Population	population density, urban, rural
	Migration	migrat*, refuge*, immigra*
	Indigenous	Indigenous, native, Maya, Inuit
	Ethnicity	ethnic*
Water issue	Scarcity or distribution	lack access, scarc*, unequal manage*
	Water quality	river quality, groundwater pollut*
Livelihoods and industry	Income	income, salary, revenue
	Farming	agricultur*, farm*, harvest*, food
	Herder	herd*, pastora*, rangeland
	Forestry	forest*, woodland, timber
	Dam	hydroelectric, irrigation dam
	Mining	mine, mining, mineral, quarry
Climate change	Extremes	flood, drought, natural disaster
	Climate variability	climate change, precipitat*, temperature
Conflict	Violent	armed, militar*, hostage, dead*, blood*
	Water conflict	water conflict, water-relate* dispute
	Resource conflict	resource* conflict, land-use conflict
	Non-violent	non-violen*, peaceful, unarmed
Cooperation	Cooperation	coopera*, collabora*, alliance, treaty
	Conflict avoidance	resolut*, negotiat*, de-escalate
Scale	Transboundary	international, global, cross-border
	Internal	intergroup, interstate, local

To analyze regional, internal, and transboundary conflict characteristics, the factors covered and the relationships between them were synthesized per continent and conflict type (studies focusing on internal, transboundary, or both conflict types). To create a geographic overview map of all studies, each study was allocated a location best representing the study area and its spatial coverage considering four different scales: continent, region (geographical regions, such as the Middle East, major river basins, or other defined larger regions), country, or local site (e.g., a city, a dam, a minor river basin, or a region within a country). In cases where there were overlaps between different regions, such as for the part of the Nile Basin also in Eastern Africa, regions were split and summed so that the total count for the overlap could be used. Locations too close to be distinguishable as separate regions/areas on a global map were merged but kept within the associated country. The map uses shapefiles of countries (Global Administrative Areas 2018), hydrological basins (Product of the Transboundary Freshwater Diplomacy Database, College of Earth, Ocean, and Atmospheric Sciences, Oregon State University 2018; The World Bank 2019), the Himalaya (Liu and Zhu 2022), and the Andes (Romano 2017), while other regions were formed by combining countries. Locations were assigned latitude and longitude coordinates using the online tool Latitude.to. Historic water conflict events were added to the map using the water conflict and cooperation database created in (Kåresdotter et al. 2023) to allow for comparison between the spatial distribution of studies and previous conflict events. Only studies focusing on one continent or one conflict type were considered in the textual analysis per continent or conflict type, meaning that 16 studies covering more than one continent and 46 studies including both internal and transboundary conflicts were excluded, respectively.

## Results and discussion

### Spatial distribution of studies

**Fig. 2:**
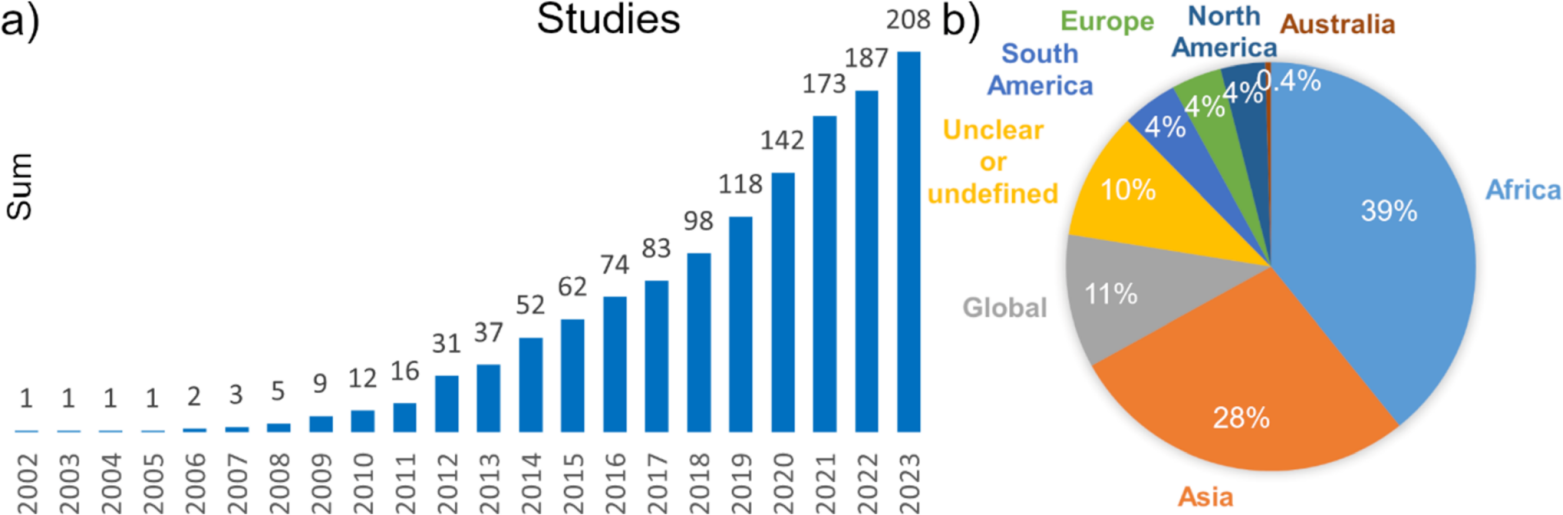
(**a**) Numbers of reviewed water conflict studies published annually in the period 2002–2023. (**b**) Relative distribution of study locations in terms of continents in the reviewed articles

**Fig. 3:**
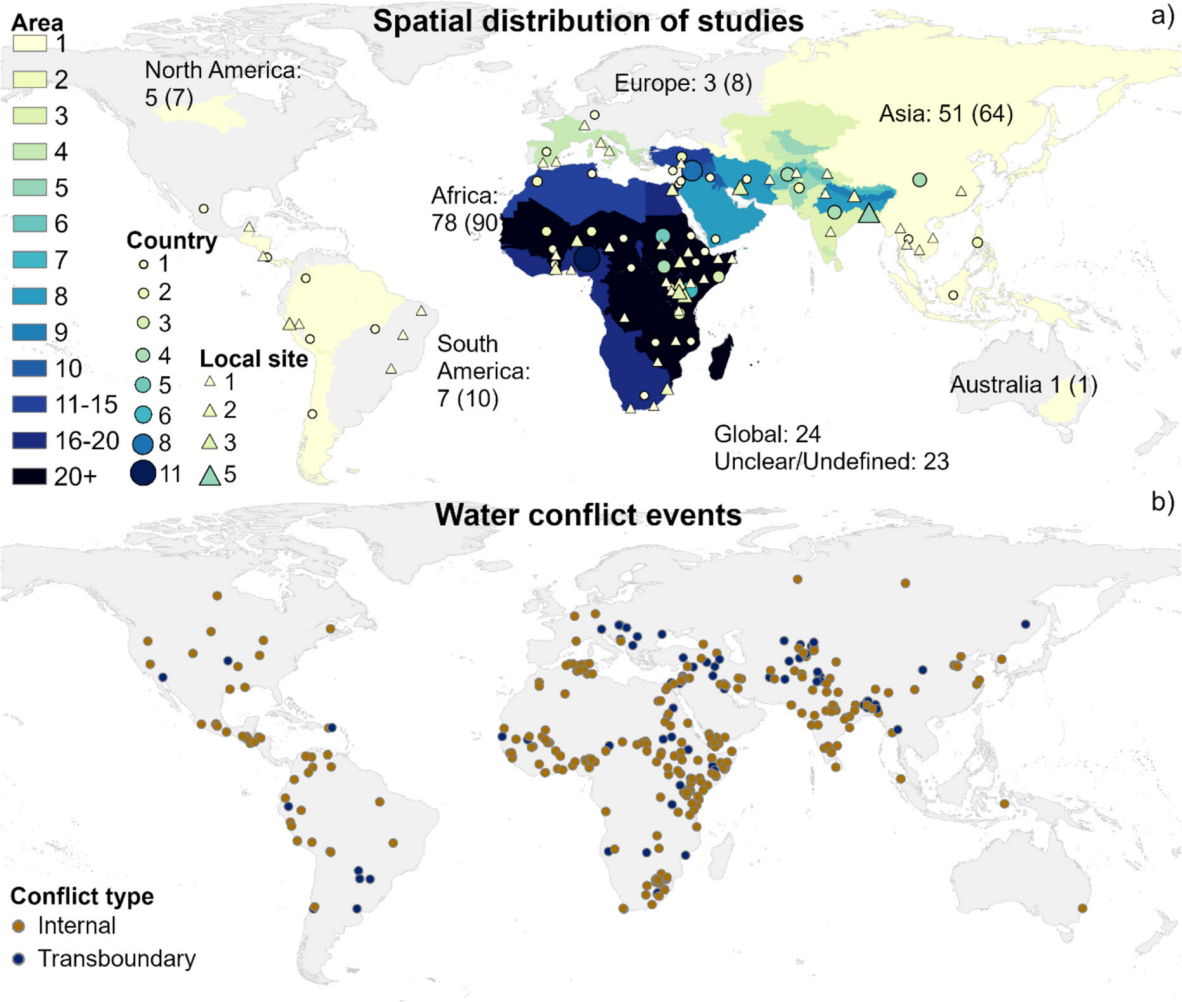
(**a**) Geographical location of studies included in this review, where the color (see key) indicates number of studies. Continents and regions have the entire area colored (overlaps are added together to form a sum), while countries (circles) and local sites (triangles) have their symbol colored and sized depending on the number of studies. Text for continents shows number of studies exclusively investigating that continent, with the total number of studies on areas within that continent in parenthesis. (**b**) Conflict events show approximate locations of internal (brown) and transboundary (dark blue) water conflicts between 1951 and 2019 from Kåresdotter et al. (2023)

Water conflict as a research topic has gained increasing interest in the past decade, with increasingly more studies published over time (Fig. 2a). After factoring in the increase in total annual number of scientific publications to date, there was still a greater relative increase of water conflict studies (using the same criteria as the review), especially since 2019, than of studies addressing the related topics of climate change and hydrology (EBSCO host, peer-reviewed academic journals). In terms of location, Africa and Asia were the most commonly investigated continents in the dataset (67% of studies), with specifically Eastern Africa, the Sahel, the Middle East, and areas around South Asia in particular being frequently represented (Figs. 2b and 3). Some studies focused on a very limited geographical location, such as a lake or a mine, while others focused on continental or global scale. The focus has more commonly been on country or local site level (54%) than on region (28%), continent (5%), or global (13%) level. The geographical distribution of conflicts in the dataset is consistent with the fact that conflict databases such as the UCDP/PRIO dataset (Gleditsch et al. 2002; Davies et al. 2022), the Transboundary Freshwater Diplomacy Database (2010), and Water Conflict Chronology (Pacific Institute 2022) show that 75–82% of reported conflicts so far have occurred in Africa and Asia. However, in direct comparison with the geographical distribution of reported water conflicts (Kåresdotter et al. 2023), shown in Fig. 3b, gaps are still found in the spatial coverage of the reviewed studies, with water conflicts in areas such as North America, South America and parts of Asia outside the Middle East and South Asia generally not being addressed or being addressed in just a small number of studies. Internal conflicts (63% of studies) were also more frequently investigated than transboundary ones (15% of studies), which is reasonable given that internal conflicts account for roughly two out of three reported water conflicts (Kåresdotter et al. 2023). Studies focusing on transboundary conflicts in a single continent were only found in Africa and Asia. If regions vary in their conflict responses and conflict risks, as many have suggested (Scheffran and Battaglini 2011; Dinar et al. 2015; Abel et al. 2019), little is then known about how climate change affects water conflicts in the under-studied areas since findings from other, more well-studied areas cannot be directly generalized and transferred to the under-studied areas. The importance of not focusing solely on areas of historical conflict was pointed out by Adams et al. (2018), arguing that a wider research focus is needed to avoid bias for regions vulnerable to change.

The reviewed studies focused more on developing than developed countries, as also found previously by (Adams et al. 2018; Perliger and Liu 2022). This can affect the identification of drivers of water-related conflicts in a climate change context since income is more closely linked to natural resources in developing countries and since there is already limited access to (water) resources in many developing regions. Other studies suggest a tendency for more conflicts to occur in areas with a lower level of economic development and where livelihoods are highly dependent on natural resources, such as in agriculture or animal herding (Gilpin 2016; Ide et al. 2021). This again suggests that simply applying the same understanding of conflicts from well-studied to under-studied areas could be flawed.

### Semi-automatic text analysis of studies

**Fig. 4:**
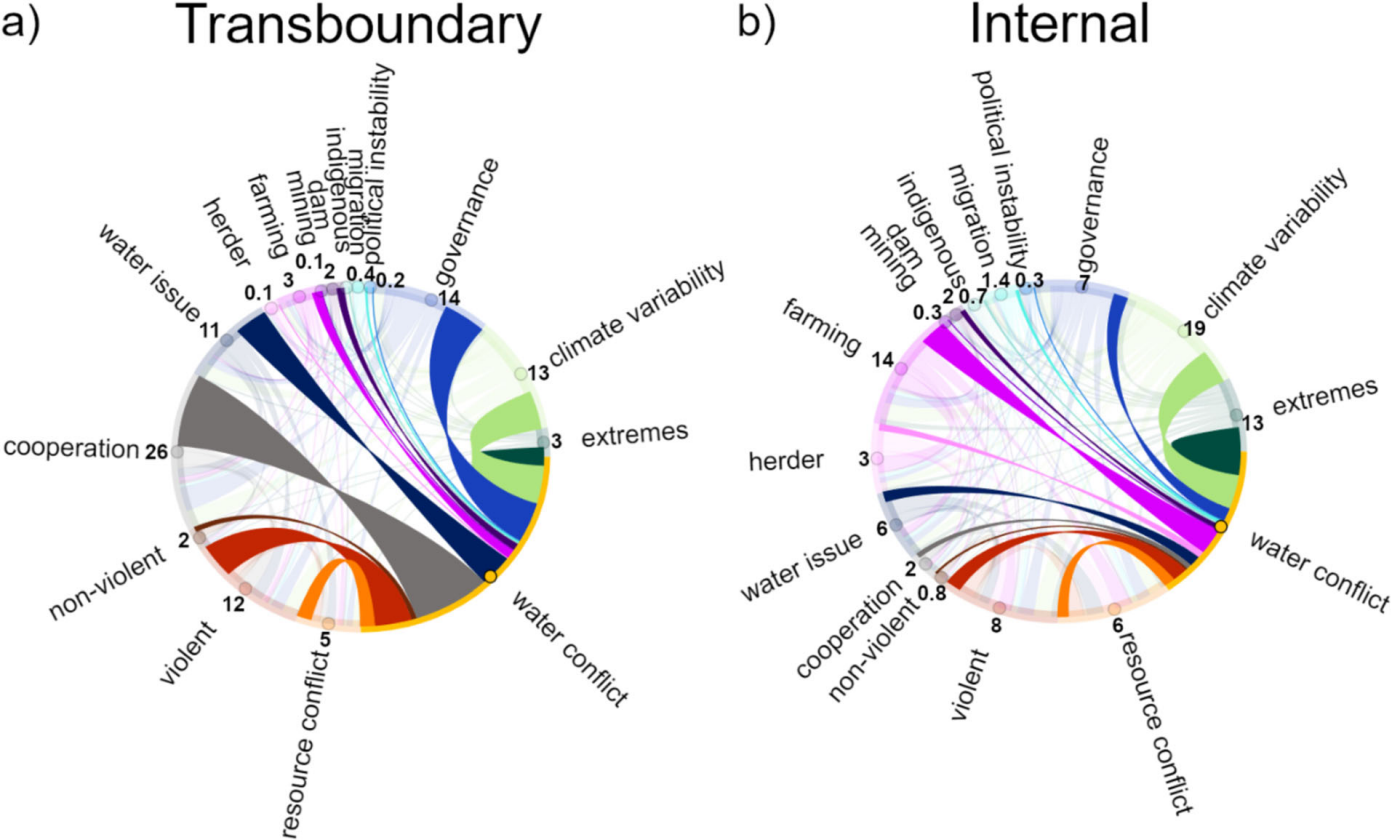
Chord diagram showing connections between water-related conflict and other concept categories, in terms of transboundary conflict studies (**a**) and internal conflict studies (**b**), where the number is the count of each connection. A score of 8.0 indicates on average 8 mentions of the concepts discussed together per article, e.g., water conflicts and governance

**Fig. 5:**
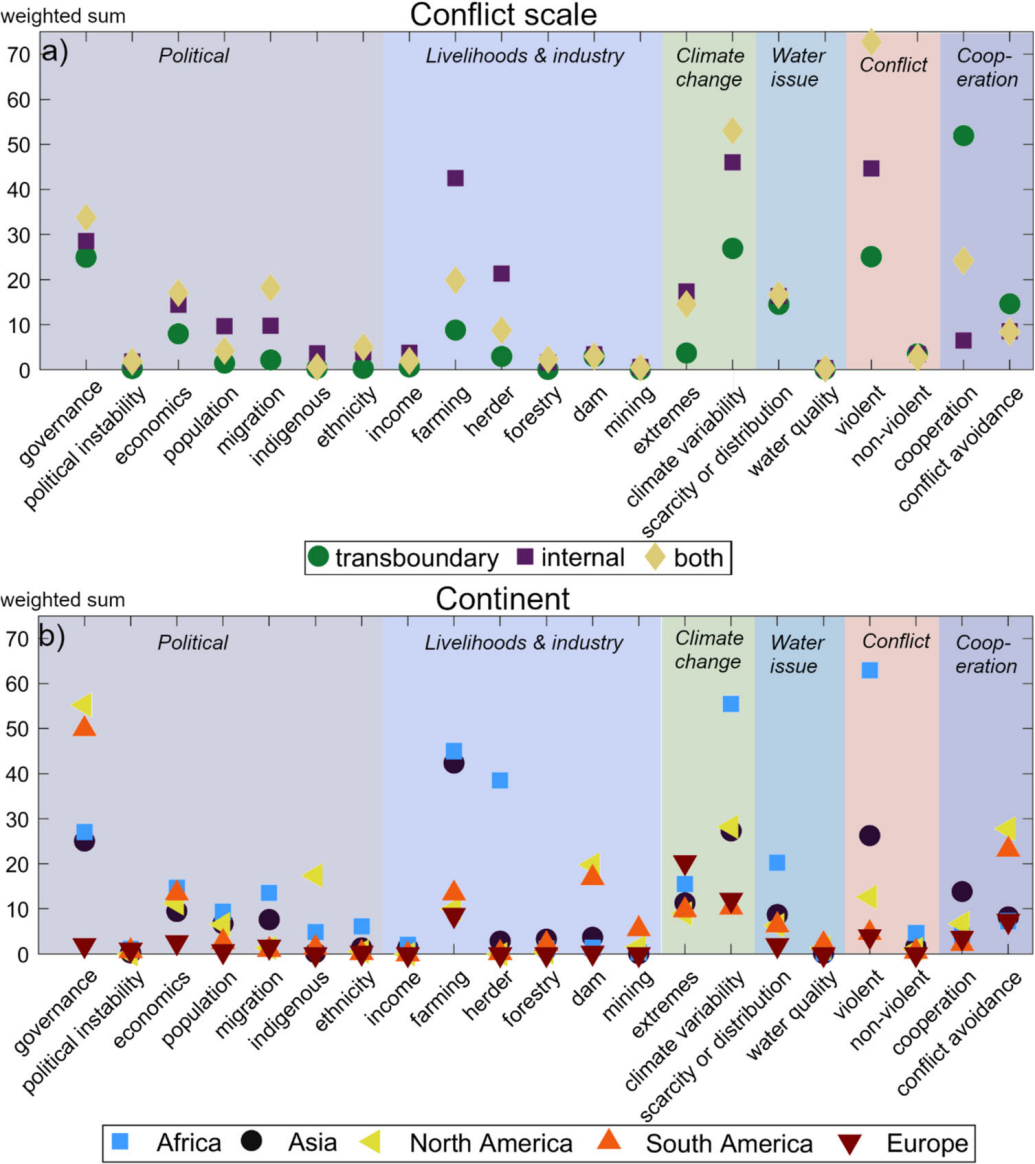
Frequency of occurrence of different concept categories (**a**) per different conflict scales and (**b**) in different continents. Average number of times a category is mentioned per article is indicated on the y-axis

Evaluating the study texts reveals common words revolving around multiple different concepts, such as governance and economics, resource management and resource scarcity, population changes and security aspects, and different scales, such as state, local, and group. Given the review inclusion criteria, the majority of the text in the reviewed studies should be relevant for climate change-affected water conflicts. Comparison between text used in analysis and full text showed that the content analysis method (evaluating different categories using part of the text from the studies instead of full texts) was generally able to capture the key dynamics around conflicts. Further analysis of word themes revealed large variation in what was discussed and to what extent. The climate change-related topics of climate variability and extremes, along with the political topic of governance, were the most commonly discussed concepts in the reviewed studies, with high total counts and almost all studies covering these factors (Figs. 4, 5). The words discussed relating to governance were almost exclusively focused on “*policy”, “govern*”, or “politic*”, with other concepts receiving no or very low mentions. Other often-mentioned categories include farming, violent, resource conflicts, conflict avoidance, transboundary, and internal. However, the categories with the highest count scores are mainly derived from higher scores for these categories in studies from Asia and Africa. It has been previously suggested that conflicts around water relate to (mis)management of the resource and not necessarily to shortage (Petersen-Perlman et al. 2017; Lopez Porras et al. 2019), which could explain why “scarcity or distribution” is widely mentioned in most of the reviewed studies, as also evidenced in that areas with a history of water scarcity also tend to have many cooperation events (Kåresdotter et al. 2023). Interestingly, there was not much mention of non-violent conflicts for any region, even though the majority of water conflicts are non-violent (e.g., 94% of conflict events included in the Transboundary Freshwater Diplomacy Database (2010)).

Looking at differences in scale (global to local, not shown) revealed that studies focusing on the global level focused more on cooperation and water management, while local site studies focused more on farming. Comparing findings between conflict scales shows different patterns in discussion between studies focusing on transboundary and internal scales (Fig. 4), indicating potential differences between these conflict types. Cooperation often involves multiple countries (Transboundary Freshwater Diplomacy Database 2010), which could explain the higher scores for cooperation categories in transboundary studies. Further investigation (Fig. 5a) revealed that internal conflicts or a combination of both types received the highest scores in all categories apart from cooperation and conflict avoidance. The tendency toward higher scores in internal conflicts could indicate higher importance or commonality in internal conflicts. However, considering similar categories receiving the highest scores for internal and transboundary conflicts, it could also mean that conflict factors have received less focus in transboundary studies, instead focusing more on cooperation which was mentioned around 50 times per study in comparison with a score of 25 for the next largest categories, namely governance, climate variability, and violent. Transboundary studies focused almost exclusively on Asia, Africa, and Global scales.

### Regional differences

**Fig. 6:**
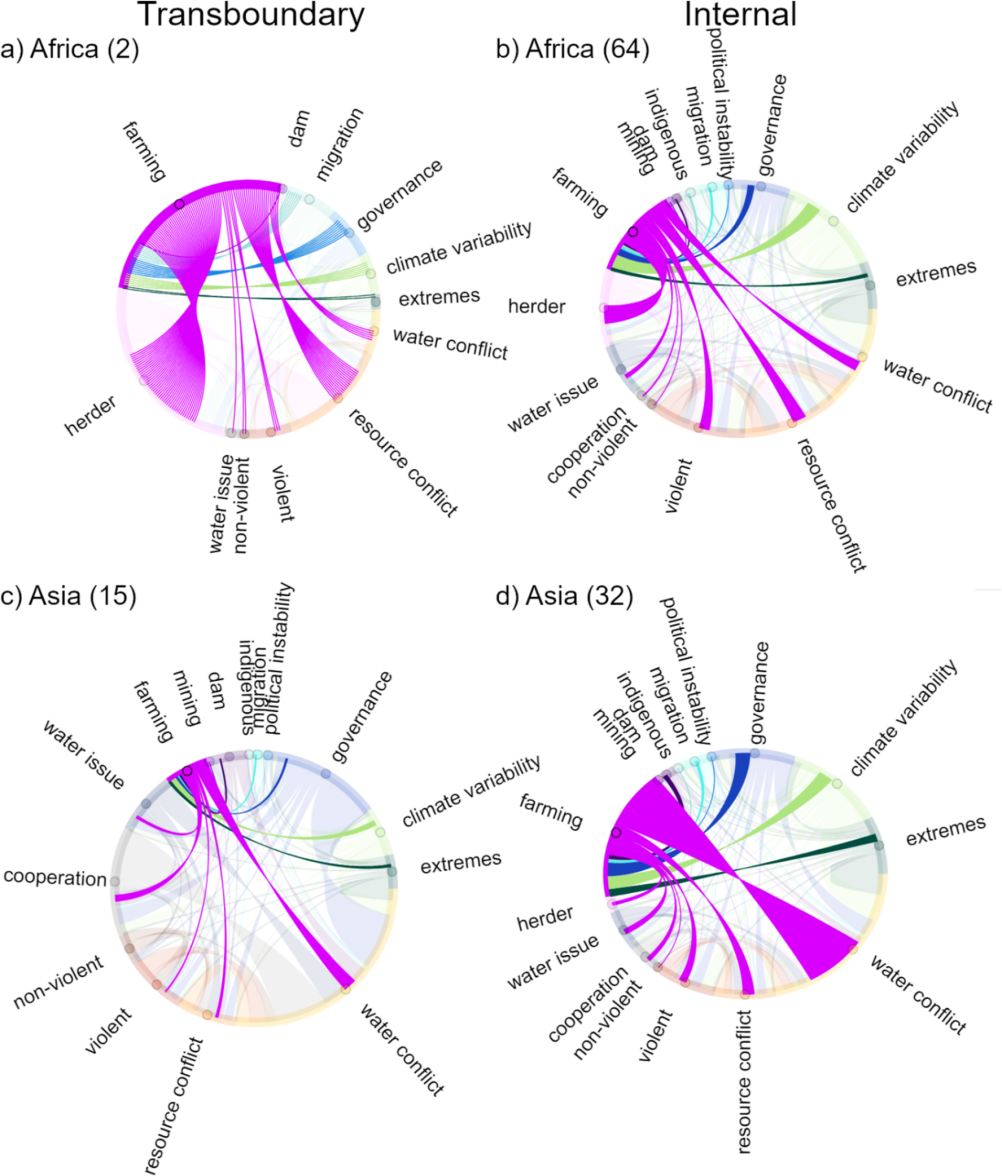
Chord diagram showing connections between farming and other categories in transboundary and internal studies in (**a**) and (**b**) Africa and (**c**) and (**d**) Asia. The number in parenthesis indicates the number of studies included per continent for each conflict type

The reviewed studies provide interesting insights into research differences in regional focus (Fig. 5). It is expected that climate change is discussed for all regions, given that it is part of the selection criteria. However, the extent of climate change discussion still varies between regions. The two most investigated regions, Africa and Asia, receive high scores for many categories, which is a strong indicator that they are important categories for water conflicts in these regions, as a larger number of studies generally means a broader discussion of a topic. However, at least in part, it could also reflect the history of some categories attracting more research interest than others. Overall, there are differences in considered conflict factors of water conflicts in regions that depend more on natural resource availability for income compared to other parts of the world, indicated by farming showing the highest scores in Asia, Africa, and South America, with herding also playing a part in Africa. As a large portion of the global freshwater use is for agriculture, up to around 90% in lower income countries (Fujs and Kashiwase 2023), it seems logical that farming stands out as an important factor in climate-affected water conflicts. As indicated in Fig. 6, the discussion around farming relationships to other factors varies depending on location and conflict type. In Africa, violent conflicts between farmers and herders are reported to be a common form of water-related conflict (Kåresdotter et al. 2023), and this theme also emerges from the reviewed studies where farming and herding are given similar scores and are largely also discussed together as shown in the chord diagram (Fig. 6a, b). The shifts in herder migration patterns could potentially also explain why Africa received the highest migration score (blue squares in Fig. 5). In Asia, governance, farming, internal, and violent were strong themes in the reviewed studies (black circles in Fig. 5b), with farming showing a fairly strong connection with governance with a score of 5 (Fig. 6b), which could indicate that water management and governance relating to agriculture are important parts of water conflicts in Asia in the studies included in the analysis. Given that dams are commonly mentioned in texts on water conflict events (Kåresdotter et al. 2023), it is surprising that there is little discussion relating to dams in Asia. This could mean that water conflicts in Asia are more affected by other factors despite significant water flow effects by dams in this region (Kåresdotter et al. 2022), that dams are not commonly investigated in climate–water–conflict nexus research, and/or that the text analysis is not picking up all dam-related terms.

For the least studied continents, namely North America, South America, and Europe, having just a few studies included means that caution is needed when interpreting the results. North America received the highest score for Indigenous, which could reflect a higher awareness of conflicts related to indigenous rights, but in this case, this resulted from one study investigating whether rainfall affected conflict in the Classic Maya civilization. For South America, law and water governance was the focus of several articles, which likely impacted the scores for governance and other political factors in this region. South America has also seen an increase in the number of conflicts, where about half of the water conflicts happened in the last 10 years of records in Kåresdotter et al. (2023), making it interesting to dig deeper into what is causing this increase. In this review, ten studies focused on South America, and almost all were published in 2019 or later, indicating an increased interest. Europe received the highest score for extremes, which is likely resulting from two out of the three papers focusing on drought. Investigation of Europe could highlight potential paths to conflict mitigation, as Europe has had no water conflicts since 2008 (Kåresdotter et al. 2023). These examples highlight the need for more studies in these regions to gain a better understanding of the climate–conflict factors that are of importance to these areas. In some areas of the world, including the USA, climate change is still a debated topic. Overall, there is large variation between countries in news coverage of this topic, the magnitude of the threat, the source, and solutions (Tschötschel et al. 2020; Pew Research Center 2022), which could also affect research publications and how related conflicts are framed and investigated in these.

Although cooperation was not the focus of this review, the fact that conflict avoidance has a higher score, while cooperation shows a low score in North and South America could indicate that these continents approach peacekeeping through conflict resolution and mediation rather than collaboration. North America and South America are also the regions where conflicts were more tied to dams. Given how strongly dams can influence water flows as well as benefits derived from the river (Lehner et al. 2011; Stephens et al. 2021), it is interesting to note that, overall, dams did not stand out as a widely considered factor in research.

## Limitations

Caution is needed when interpreting the findings from the text analysis and the links between different categories in the reviewed studies. Given the focus on climate change as part of the inclusion criteria, it would be wrong to conclude from the studies that climate change is driving water conflicts. Furthermore, finding no relevant studies or no connections between categories does not necessarily mean that there is a research gap as, e.g., relevant studies might exist but were not found using the search criteria. Similarly, a strong connection between two categories or a high score for a concept indicates much discussion around the topics but does not necessarily mean a strong relationship in water conflict studies. The spatial gap in studies also shows that some conflicts have not been covered in the existing research. Few studies in a region lead to uncertainty in results, as the topics covered in one study often are limited, and a wider range of studies from different fields could be needed to cover the factors involved in various conflict events fully. That said, focusing on the parts of the studies mentioning words relating to conflict and drivers, as done in this review, meant that the categories were likely mentioned in a relevant context and most likely in a relevant discussion on conflict, as indicated by the similarities between context texts and categories discussed together. One potential explanation for the lack of studies covering certain areas is the exclusion of languages other than English. Non-English written publications, which are common in, for example, South America, Germany, and China (Amano et al. 2021) would not be a part of this review. Including multilingual scholars and scholars from the Global South in research projects could help address this limitation and provide a more comprehensive view of the topics across different linguistic and cultural contexts.

Transboundary and internal conflicts showed different results, which is reasonable given that they are likely to have different characteristics. However, the complex relationships linking internal and transboundary factors in conflicts mean that these conflict types can be connected and affect each other. For example, migration from conflict areas can increase conflict risk and upstream use of water can cause local conflicts downstream. Therefore, to fully understand a conflict, analysis should include different spatial scales.

A normalized weighted average was used to make results more directly comparable between regions, with the inclusion of more studies leading to greater diversity and heterogeneity of categories and factors considered among them. This is reasonable since an individual article would generally not discuss all categories considered in this review but rather focus on one or a few categories. For regions or conflict types with fewer studies, such as South America and transboundary studies, this can affect the results, leading to some concepts potentially appearing more important than if a larger number of studies were included for that region. Such effects could explain why Africa has a more diverse range of connections compared to Europe, where the conflict discussion is linked largely to climate change extremes. This emphasizes the need for more studies of so far under-studies topics and areas of the world to broaden our understanding of water-related conflict issues in those areas, which may, e.g., include categories less commonly prevalent and studied for other parts of the world, such as issues related to Indigenous people. However, the lack of regional studies of a specific category does not necessarily reflect a research gap but could instead reflect an absence of problems relating to that category in a region. Further investigating into historic conflict events previously not investigated can help provide insights into relevant factors. It should also be noted that the use of a single search engine and a semi-automated procedure for study inclusion risks excluding studies that may, in principle, fit the study aims.

## Conclusions

Studies investigating the climate–water–conflict nexus have been limited in their spatial coverage, with most studies focusing on Africa and parts of Asia. This could, in part, be explained by the fact that most historic water conflicts have taken place in these parts. However, freshwater availability is affected by changes in human impacts and climate, which give rise to potential water conflicts in regions less researched. Increased instances of conflict in South America appear to have caused increased research interest. However, studies are still limited in their scope, and more knowledge is needed to understand the factors behind this increase. Collaboration with multilingual and local researchers could help locate non-English publications to understand better the underlying factors affecting these conflicts and how they could be addressed. Developing countries have received the most research interest, but other regions, such as North America, have also had recent water conflict events. Digging deeper into how climate, politics, and other factors could give clues to how these conflicts will evolve, and potential differences compared with developing countries’ conflict pathways. Further, an investigation into what factors have been of importance to explain why Europe has had no water conflicts since 2008 could highlight potential paths to conflict mitigation that can be adapted and used in other regions to limit future conflicts.

Besides climate change, the most widely discussed concepts in the 208 water conflict articles reviewed in this study were related to political factors and livelihoods and industry, with findings relating to farming and dams being the most prominent. Climate change was an inclusion criterion and, as such, widely discussed in the reviewed studies, likely more as a threat multiplier than a main driver of conflict, as indicated by the number of other factors appearing as important. For continents, no single variable emerged as the main driver in any continent, indicating that multiple aspects together form conflict pathways. The same is largely true when comparing studies focusing on transboundary and internal conflicts, apart from cooperation, which is widely addressed in transboundary-focused studies. For example, transboundary conflicts are more largely connected to political factors, while internal ones are more connected to livelihoods and resource use. How water is managed, however, could be a relatively general key to understanding and preventing conflict. More specifically, farming is important in studies of water conflicts in Asia, while conflict studies in Africa also often consider issues between farmers and herders. Internal conflicts emerge as more commonly investigated, which is reasonable given that they account for roughly 2/3rd of reported water conflicts (Kåresdotter et al. 2023). Conflict factors could be of varied importance depending on whether the water conflict is internal or transboundary, as indicated by differences between studies focusing on these conflict scales. Due to a limited number of studies outside of Africa and Asia, it is difficult to draw robust conclusions regarding conflicts in the under-studied other parts of the world. The reviewed studies of other continents were predominantly focused on a few specific conflict subjects, which significantly influenced the outcomes and made it difficult to generalize the results. Consequently, there is a critical need for more research to cover a more widespread range of regions and more diverse subjects to gain a more comprehensive understanding of water conflicts globally. While local, political, and cultural contexts can combine into different pathways to conflict, this review indicates that political factors, livelihoods and industry, along with climate change that was required for inclusion, are generally important aspects to consider in water-related conflict. However, as political contexts differ widely across the globe, further investigation is needed into regional differences in aspect importance for different parts of the world, to provide more insight into conflict pathways. For example, in parts of the world with a history of conflicts, incomes are more closely linked to natural resources and access to water. Having access to income insurance and/or unemployment benefits has been found to reduce the likelihood of conflict (Miguel et al., 2004; Abel et al. 2019) and is one political factor that can differ widely around the globe.

Future research should give priority to so far under-studied areas, such as South America and parts of Asia outside the Middle East, and South Asia and pay special attention to areas identified as particularly vulnerable to future hydroclimatic changes to create the knowledge needed to limit future security risks. Recognizing non-English research and publications and promoting partnerships with Global South scholars is important to this knowledge building. With the help of more automated text analysis methods, such as text extraction and text mining approaches used in this review, previous conflict events and new studies and reports could be further analyzed in a timely manner, adding information from other sources, such as news media and governmental reports.

To develop effective policies and chart the course for future regional development, it is important to have a clear understanding of the key conflict factors that operate within a particular region. This study has shown that governance and policy development play an important part in regional conflicts. Knowing important regional conflict factors helps policymakers to assess the region’s strengths and weaknesses, identify areas of potential growth, and develop policies that are tailored to the unique needs of the region. Therefore, understanding the regional conflict factors is a crucial first step toward ensuring informed, effective, and sustainable policy development. Research is especially needed on the topics of cooperation, non-violent conflicts, and future projections, as knowledge of these will be vital in understanding and creating recommendations that can enable conflict mitigation and prevent future water-related conflicts.

## Data Availability

A list of all included studies and the words used to form each category can be downloaded in Zenodo: 10.5281/zenodo.8383299.
